# Rapid Prototyping of Anomalous Reflective Metasurfaces Using Spray-Coated Liquid Metal

**DOI:** 10.3390/ma17092003

**Published:** 2024-04-25

**Authors:** Glan Allan V. Manio, Matthew T. Kouchi, Saige J. Dacuycuy, Aaron T. Ohta, Wayne A. Shiroma

**Affiliations:** Department of Electrical and Computer Engineering, University of Hawai’i at Mānoa, Honolulu, HI 96822, USA; mkouchi2@hawaii.edu (M.T.K.); saiged@hawaii.edu (S.J.D.); aohta@hawaii.edu (A.T.O.)

**Keywords:** liquid metal, metasurface, anomalous reflection, Galinstan

## Abstract

Reconfigurable intelligent surfaces (RISs) have the potential to improve wireless communication links by dynamically redirecting signals to dead spots. Although a reconfigurable surface is best suited for environments in which the reflected signal must be dynamically steered, there are cases where a static, non-reconfigurable anomalous reflective metasurface can suffice. In this work, spray-coated liquid metal is used to rapidly prototype an anomalous reflective metasurface. Using a pressurized air gun and a plastic thin-film mask, a metasurface consisting of a 6 × 4 array of Galinstan liquid–metal elements is sprayed within minutes. The metasurface produces a reflected wave at an angle of 28° from normal in response to a normal incident 3.5-GHz electromagnetic plane wave. The spray-coated liquid–metal metasurface shows comparable results to an anomalous reflective metasurface with copper elements of the same dimensions, demonstrating that this liquid–metal fabrication process is a viable solution for the rapid prototyping of anomalous reflective metasurfaces.

## 1. Introduction

Reconfigurable intelligent surfaces (RISs) promise to improve coverage for signals negatively impacted by obstructions present in the line-of-sight (LOS) path, making them an attractive technology for 5G and beyond mobile communications [1]. A typical RIS combines passive reflective elements with active devices such as varactors [2,3,4,5] or PIN diodes [6,7,8,9], while others use phase-tunable materials such as liquid crystals [10,11,12], 2D materials [13,14,15,16,17], or phase-change materials [18,19]. Creating a tunable reflection phase gradient across a surface enables the RIS to dynamically redirect an incoming signal to an anomalous direction not predicted by Snell’s Law.

**Figure 1:**
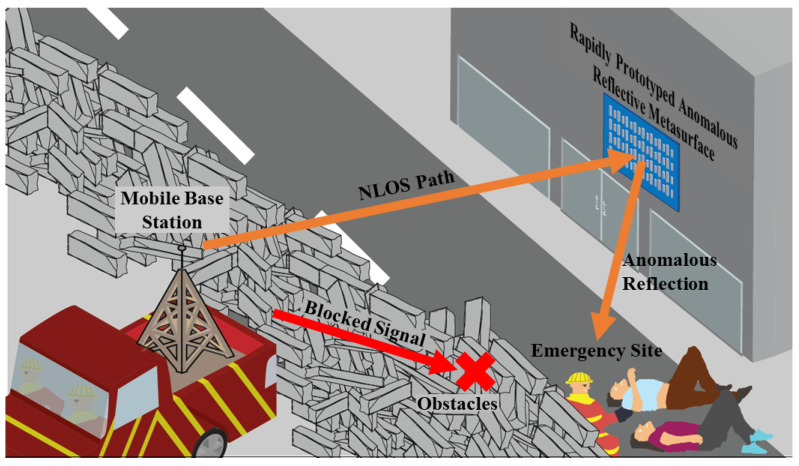
A rapidly prototyped anomalous reflective metasurface provides an NLOS link between a mobile base station and an emergency site that otherwise has a blocked LOS path.

In some use cases, however, an RIS may require little to no reconfiguration of the reflected signal [20]. For example, in the aftermath of a natural disaster, a rapid-response network can be set up, as shown in Figure 1. Here, a mobile base station is deployed to an emergency worksite, but obstacles block a LOS link from being established to emergency workers aiding survivors. Under such conditions, where the emergency workers are relatively stationary for hours at a time, a static anomalous reflective surface with little to no reconfigurability is sufficient. The reflective metasurface can be rapidly prototyped and positioned to provide an anomalous-angle non-line-of-sight (NLOS) link to the emergency workers. If the emergency workers subsequently move to a different site, say to the top left of Figure 1, another rapidly prototyped metasurface can be quickly fabricated using a different template that provides the appropriate anomalous reflection angle.

Although a static anomalous reflective metasurface is limited to a fixed reflection angle, it is simpler and less expensive than an RIS with active elements. Metasurface geometries have previously been created with copper elements using etching, milling, or placing copper tape on an unclad substrate [21]. Other metasurfaces have been explored using sputter deposition [22], inkjet printing [23], screen printing [24,25], and 3D printing to create reflective elements [26]. Although these fabrication methods are effective, spray-coated liquid metal for rapid prototyping has numerous advantages compared to traditional fabrication methods. The main components that differentiate our proposed method are portability, simplicity, and scalability. Compared to methods such as copper milling/etching, 3D printing, and inkjet printing, spray-coating provides a faster fabrication time with less expensive equipment. The proposed method also has a simple fabrication process, only requiring the creation of a mask and spray-coating the liquid metal onto a substrate. Once the mask is created, spray-coating of liquid metal has a faster scalability implementation, where the mask can be moved to new locations for the addition of reflective elements. Therefore, an alternative rapid prototyping method utilizing spray printing of liquid–metal metasurface elements is investigated.

The liquid metal used here is Galinstan (Rotometals Incorporated, San Leandro, CA, USA), a near eutectic gallium-based alloy consisting of Ga 68.5 wt%, In 21.5 wt%, Sn 10 wt%. Galinstan, with this composition, has a melting temperature of 11 °C [27]. Due to the adhesiveness of Galinstan occurring from the oxide skin produced by the spray deposition, electrolytes such as sodium hydroxide (NaOH) can be used to remove Galinstan after use and preserve the substrate material for reusability [28,29]. Galinstan also has a high electrical conductivity of 3.46 × 10^6^ S/m [30], making it an attractive metal for the aerosol spray deposition of conductive films [31,32].

Although spray deposition using conductive paint has been previously demonstrated for transmissive metasurfaces [33], this paper reports the first rapidly prototyped aerosol spray deposited liquid–metal anomalous reflective metasurface. Using a pressurized air gun and a plastic thin-film mask, liquid metal is patterned on a substrate, with an array of liquid–metal elements capable of being sprayed within minutes. Successful tests were conducted, demonstrating the viability of anomalous reflective metasurfaces fabricated using the rapid prototyping method.

## 2. Materials and Methods

One of the defining features of an anomalous reflective metasurface is its ability to induce reflections at an angle that is not predicted by the conventional Snell’s Law. This is made possible by engineering a phase gradient on the surface between two media, which, in turn, is generated by periodically arranging unit cells with varying geometries across the surface [34]. To quickly apply these geometries to a surface, spray deposition of liquid metal is utilized.

### 2.1. Unit Cell Design

**Figure 2:**
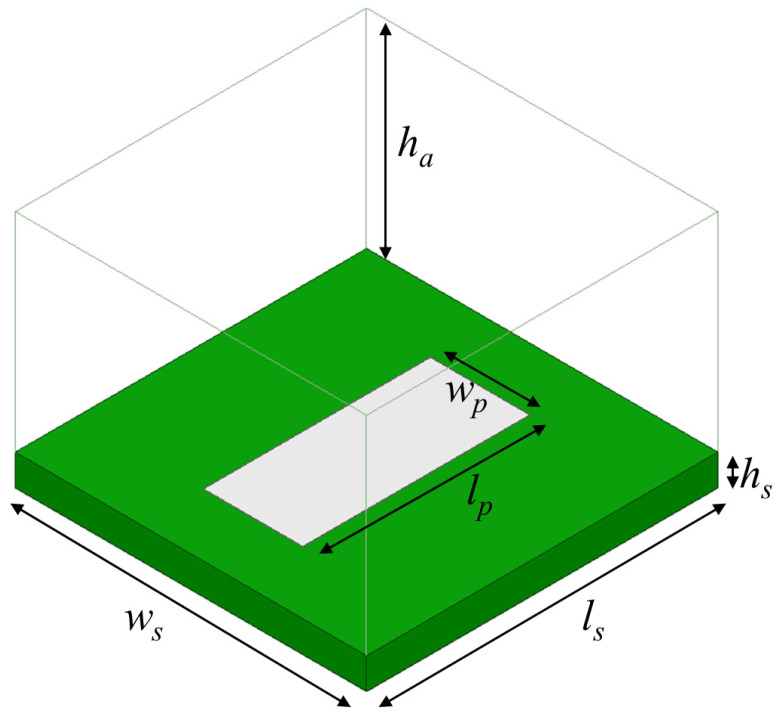
A 3.5-GHz unit cell with *h_a_* = 21.4 mm, *h_s_* = 3.175 mm, *w_s_* = 36.3 mm, *l_s_* = 36.3 mm, and *w_p_* = 10.3 mm. The patch length *l_p_* was set to one of the following values: 26.5, 23.5, 29.5, or 27.5 mm, depending on the desired phase of the reflected signal. The patch height *h_p_* was 35 μm, but this dimension is not visible in this figure. A Floquet-port simulation mimics an infinite array of this unit cell geometry.

Figure 2 shows the metasurface unit cell, which consisted of a rectangular Galinstan element (length *l_p_*, width *w_p_*, height *h_p_*) on a Rogers *RT/duroid* 5880 substrate (*h_s_* = 3.175 mm, *ε_r_* = 2.2, tan *δ* = 0.0009) (Rogers Corporation, Chandler, AZ, USA). This substrate was specifically selected because of its low-loss characteristics to enhance the reflective metasurface efficiency. A 35.6-μm-thick copper backplane was positioned beneath the substrate. The unit cell dimensions (length *l_s_*, width *w_s_*) largely set the operating frequency of 3.5 GHz.

The unit cell was designed in Ansys HFSS, using a Floquet-port simulation to mimic a normally incident uniform electromagnetic plane wave source on an infinite array of these unit cells. The air-box height *h_a_* was set to λ_0_/4, although de-embedding was enabled to simulate the reflection coefficient *S*_11_ at the surface. The key to designing an anomalous reflective metasurface is to vary the geometry within a given unit cell—thereby resulting in a distinct *S*_11_ state—and then positioning different unit cell states in a quasi-periodic pattern that yields a phase gradient for the desired anomalous reflection.

**Figure 3:**
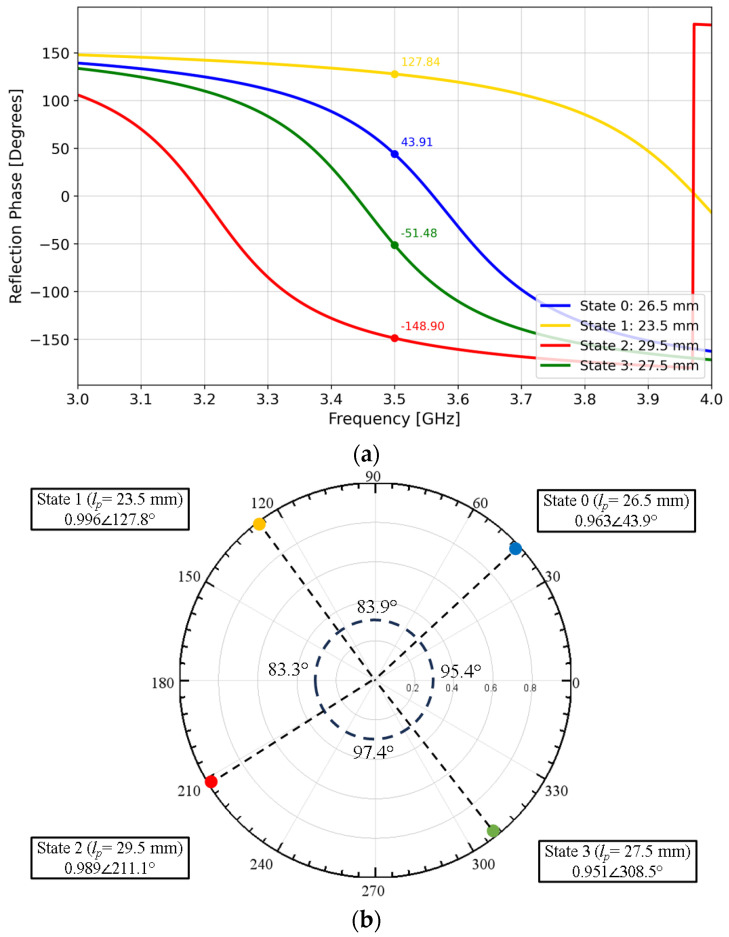
(**a**) Reflection phase versus frequency response and (**b**) polar plot of four states (States 0 to 3) with a ~90° phase shift in *S_11_* between adjacent states at 3.5 GHz.

For example, if *l_p_* is set to 26.5 mm, a Floquet-port simulation that mimics an infinite array of identical unit cells results in the blue curve of the reflection phase versus frequency response shown in Figure 3a. Repeating the Floquet-port simulation for *l_p_* = 23.5, 29.5, and 27.5 mm resulted in three additional curves (yellow, red, green).

In short, varying the patch length *l_p_* results in four distinct *S*_11_ states that are approximately 90° apart at 3.5 GHz (Figure 3b), hereafter referred to as State 0, 1, 2, and 3. An array consisting of an alternating State 0-1-2-3 periodic pattern of these unit cells then yields a 360° phase gradient in ~90° increments. This phase gradient, in turn, results in anomalous reflection as predicted by the Generalized Snell’s Law of Reflection [35]. Based on a 3.5-GHz operating frequency with a State 0-1-2-3 phase progression pattern, the anomalous reflection angle for normal incidence given by the Generalized Snell’s Law is:

(1) $$\begin{array}{lll} \theta_{r} & = & \sin^{-1}\left(\frac{\lambda_{0}}{360^{\circ }}\frac{d\phi }{dx}\right)\\ & = & \sin^{-1}\left(\frac{\frac{3\times 10^{8}m/s}{3.5\times 10^{9}\mathrm{Hz}}}{360^{\circ }}\frac{83.9^{\circ }+83.3^{\circ }+97.4^{\circ }+95.4^{\circ }}{4\times 0.0363m}\right)\\ & \approx & 36^{\circ } \end{array}$$

An anomalous reflective metasurface with four unit-cell states ~90° apart is known as 2-bit coding [36], where arranging the unit cells in the State 0-1-2-3 pattern results in a single-beam reflection. Similarly, a metasurface with just two unit-cell states ~180° apart is known as 1-bit coding. A 1-bit metasurface with a State 0-0-2-2 pattern would result in the same reflection angle θ_r_ as the State 0-1-2-3 pattern but would also produce a symmetric beam at −θ_r_ [37]. Eliminating the symmetric beam to realize a single-beam reflection would require a large oblique angle of incidence [38].

### 2.2. Array Design

The unit cell results presented above were based on an HFSS Floquet-port simulation, which assumes an infinite array. An HFSS bistatic radar cross-section (RCS) simulation was performed next, again using a periodic State 0-1-2-3 pattern to simulate a finite array.

**Figure 4:**
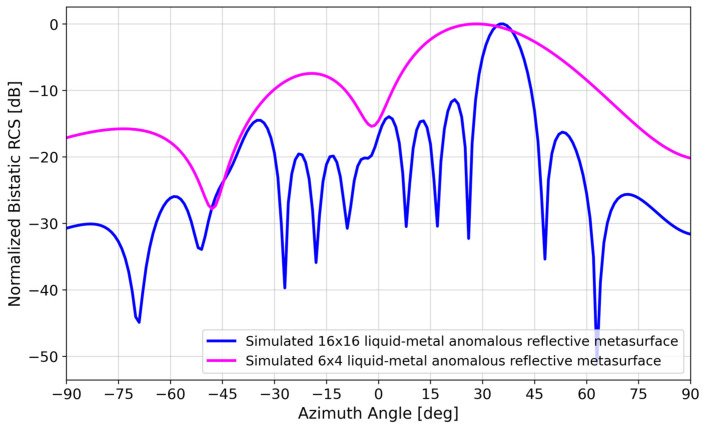
Simulated bistatic RCS of a 16 × 16 element and 6 × 4 element array to compare to Generalized Snell’s Law calculation.

Figure 4 shows the bistatic RCS of a 16 × 16 element array with a simulated reflected beam at ~36°, in agreement with the Generalized Snell’s Law calculation. Figure 4 also shows that a 6 × 4 element array results in a lower directivity as expected for a smaller array, but the ~28° reflection is still close to that predicted by Generalized Snell’s Law and has the additional advantage of requiring ~10 times fewer elements than a 16 × 16 array. The sections that follow thus focus on the smaller 6 × 4 array.

**Figure 5:**
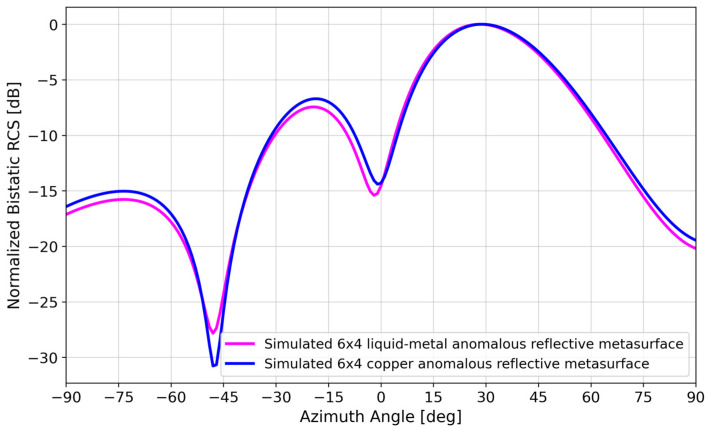
Simulated bistatic RCS of a 6 × 4 liquid–metal metasurface vs. simulated 6 × 4 copper metasurface.

Although Galinstan has a lower conductivity (3.46 × 10^6^ S/m) than copper (5.8 × 10^7^ S/m), Figure 5 shows that the reduced conductivity does not appreciably affect the bistatic RCS. This point is discussed in more detail in Section 4.

### 2.3. Fabrication

**Figure 6:**
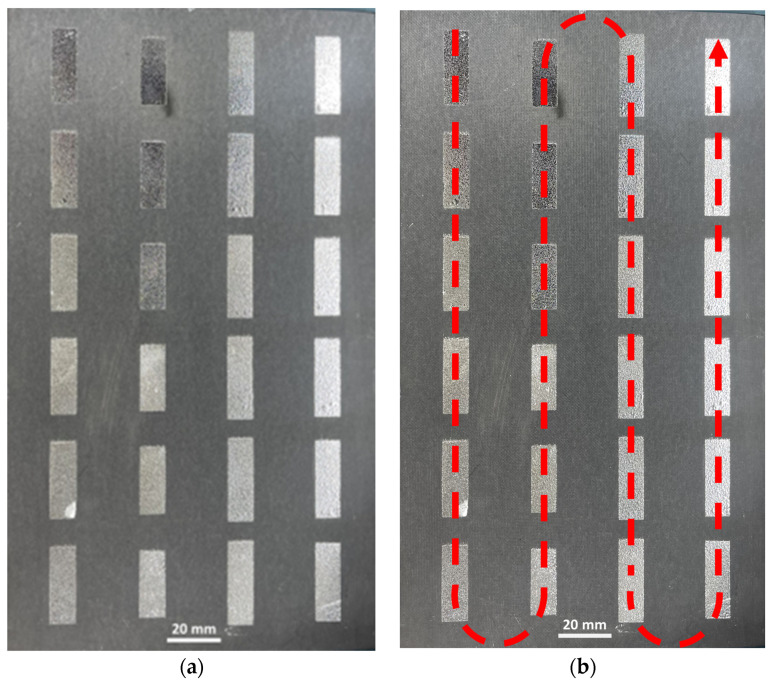
(**a**) Fabricated 6 × 4 element spray-coated liquid–metal metasurface. (**b**) Dashed line showing the approximate airbrush trajectory during the spray-coating process.

A 0.1-mm thick mylar/transparency film was used as a mask for the spray-coating process. The mask was placed on a 229 mm × 152 mm Rogers *RT/duroid* 5880 substrate, and a Master G233 commercial airbrush (TCP Global Corporation, Las Vegas, NV, USA) was used to spray-coat Galinstan liquid metal, with the airbrush nozzle positioned perpendicular to the substrate surface. To reduce oxidation of the spray-coated liquid metal, the air pressure was set to the lowest setting of 20 psi, and the airbrush nozzle was placed 5 cm from the substrate surface [39]. Each patch was sprayed for less than one second in an up-and-down S-pattern, one element at a time until all patches had an even coating of liquid metal (Figure 6).

**Figure 7:**
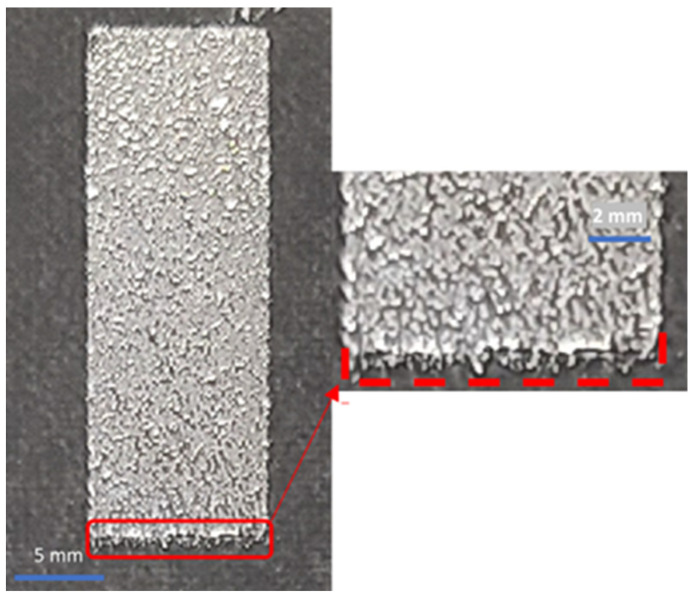
Zoomed-in image of a spray-coated liquid–metal element with under-spraying error. The dashed line indicates the as-designed dimensions of the patch.

**Figure 8:**
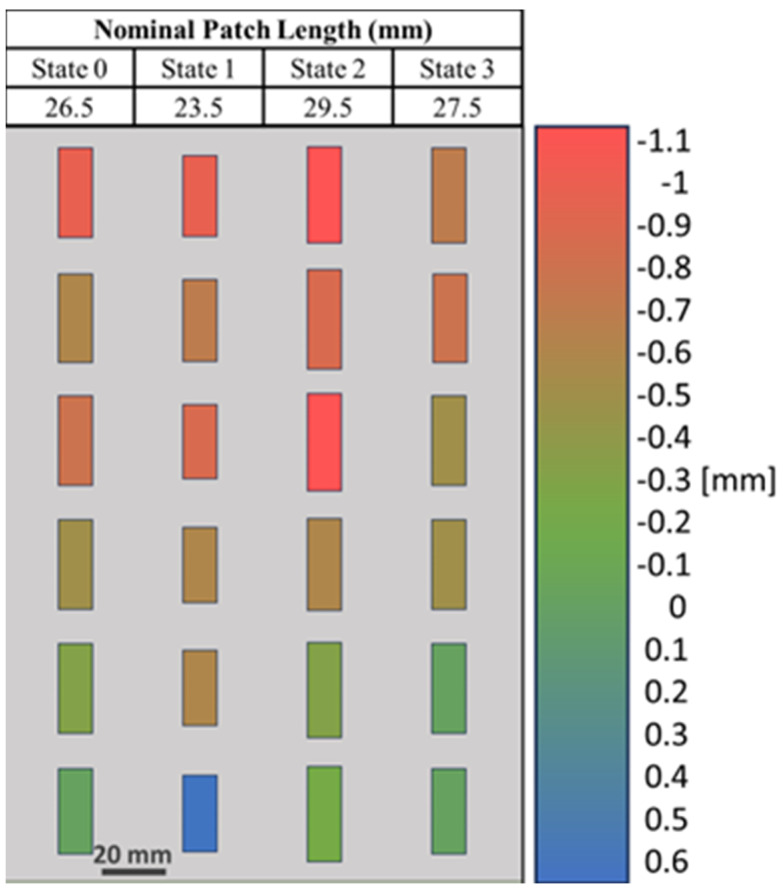
Variations in the patch lengths of the as-fabricated 6 × 4 spray-coated liquid–metal metasurface compared to the nominal as-designed patch lengths. The patch length variations from the as-designed length are indicated with a gradient heat map ranging from red (under-sprayed; as-fabricated patch was shorter than design) to blue (over-sprayed; as-fabricated patch was longer than design).

**Figure 9:**
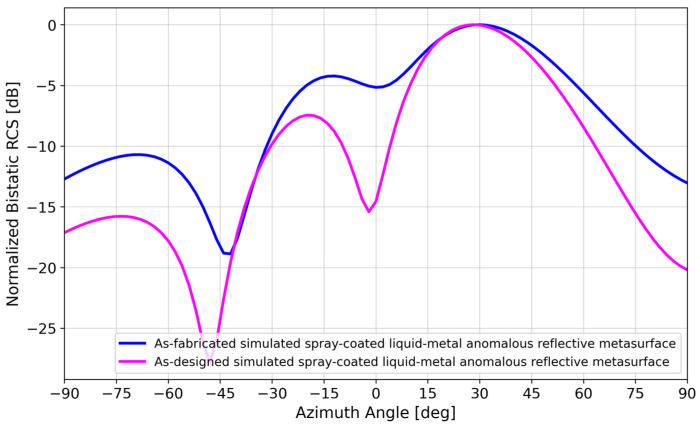
Bistatic RCS simulations of the 6 × 4 spray-coated liquid–metal metasurface using the as-designed versus as-fabricated lengths of each patch.

After fabrication, small variations in the lengths of several patches were observed (Figure 7). To investigate further, the dimensions of each of the 24 patches were measured (Figure 8). The average variation in patch length *l_p_* was 1.74%, where most of the patches exhibited shorter lengths due to under-spraying. The average variation in the patch width *w_p_* was 0.22%, which is negligible (Table S1). A simulation using the as-fabricated lengths of each patch is shown in Figure 9. A detailed explanation comparing these two curves follows in Section 4.

## 3. Results

### 3.1. Bistatic RCS Measurements

**Figure 10:**
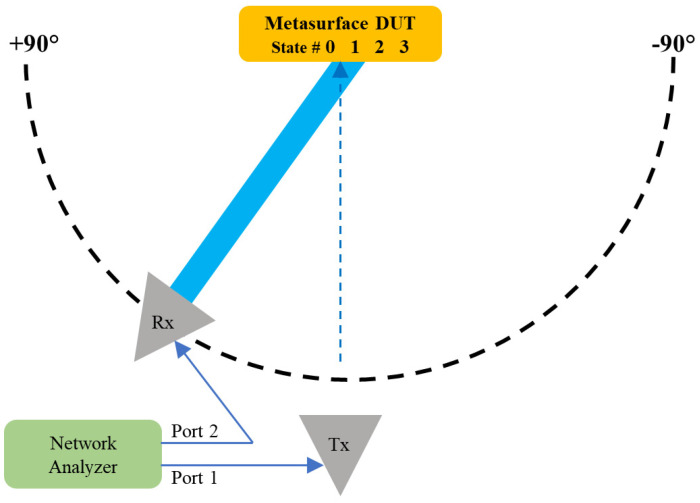
Top view of the experimental setup consisting of an anomalous reflective metasurface DUT, a transmitting horn antenna (Tx) normal to the metasurface, a receiving horn antenna (Rx) on a rotating arm, and a network analyzer.

Figure 10 shows the experimental setup for measuring the metasurface’s bistatic RCS. A 9-dBi-gain transmitter antenna was positioned for normal incidence at the boresight of the Device Under Test (DUT), while a 9-dBi-gain receiver antenna was mounted to a Newport MM3000 1-axis motion controller (Newport Corporation, Irvine, CA, USA) and rotated azimuthally about the metasurface DUT in 2° increments. A Keysight FieldFox N9951B network analyzer (Keysight, Santa Rosa, CA, USA) was connected to both antennas. To accurately measure the performance and avoid multipath interference, the experiment was conducted in a large open area outdoors with the metasurface DUT positioned in the far field of both the Rx and Tx antennas.

**Figure 11:**
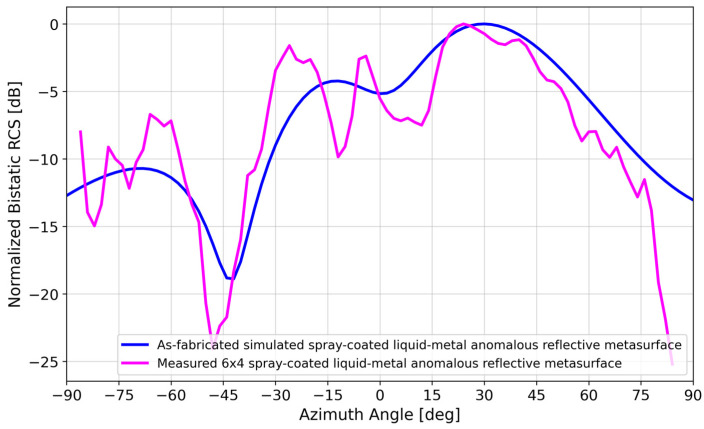
Normalized bistatic RCS of simulation vs. measurement of the as-fabricated spray-coated liquid–metal metasurface.

Figure 11 compares the bistatic RCS of the as-fabricated simulated versus measured spray-coated liquid–metal metasurface. There is good agreement, particularly at the ~28° anomalous reflection angle. This agreement is closely maintained for most of the single-beam lobe between 24 and 30°.

**Figure 12:**
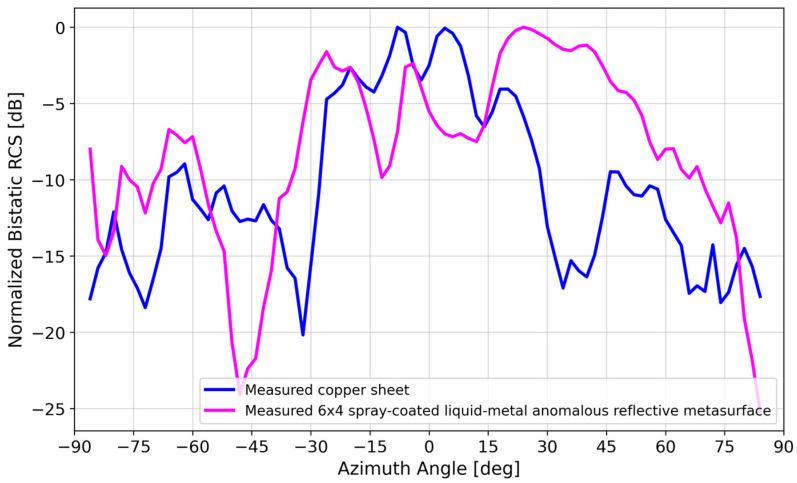
Measured bistatic RCS pattern of spray-coated liquid–metal metasurface vs. 229 mm × 152 mm copper sheet.

Figure 12 compares the measured bistatic RCS of the spray-coated liquid metal to that of a copper sheet of the same size, replacing the metasurface DUT in Figure 10. At the 28° anomalous reflection angle, the copper sheet reflected ~9 dB less power than the spray-coated liquid–metal reflective metasurface. On average, the spray-coated liquid–metal reflective metasurface performed ~10 dB better than the copper sheet within the 20°–40° anomalous reflection beamwidth.

### 3.2. Directivity Analysis

Adopting the methodology employed by [40], we compared (1) the maximum directivity of a uniform aperture of the same area as the 6 × 4 metasurface, (2) the simulated maximum directivity of the as-designed and as-fabricated 6 × 4 liquid–metal metasurface from HFSS, and (3) the measured maximum directivity of the fabricated 6 × 4 liquid–metal metasurface. Table 1 summarizes this comparison.

**Figure 13:**
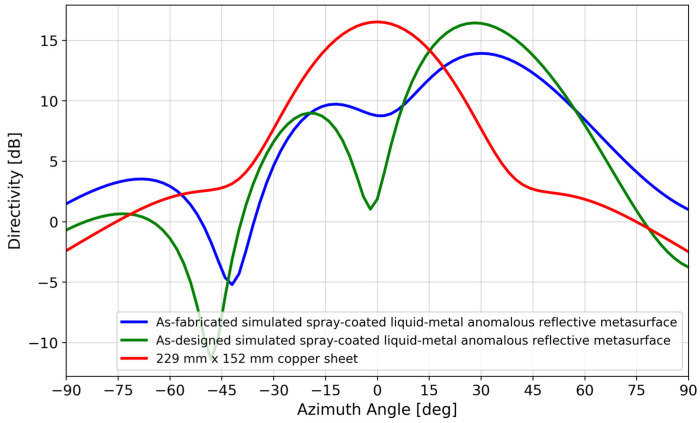
Directivity simulations of a 229 mm × 152 mm copper sheet and the as-fabricated and as-designed spray-coated liquid–metal metasurface.

For a perfectly conducting uniform aperture of the same area as the 6 × 4 metasurface (*A* = 229 mm × 152 mm), the maximum directivity is $\frac{4A}{\lambda^{2}}$ = 17.7 dBi [41]. For a liquid–metal metasurface occupying the same area, the maximum directivity was 16.4 dBi (as designed) and 13.9 dBi (as-fabricated), according to HFSS simulations (Figure 13).

The measured maximum directivity can be estimated from [41]

(2) $$D_{0}≃\frac{32400}{\Theta_{1d}\Theta_{2d}}$$

where $\Theta_{1d}{,\;\Theta }_{2d}$ are the half-power beam widths in orthogonal planes. Based on the measured data for the 6 × 4 liquid–metal metasurface in Figure 12, the maximum directivity was estimated to be 13.2 dBi after subtracting 3 dB due to the sizable minor lobe [41].

As seen in Table 1, the measured maximum directivity was 4.5 dB less than that of a comparison perfectly conducting uniform aperture. By comparison, [40] had an 8 dB difference, understandably larger given the significantly higher frequency and design complexity compared to this paper’s passive design.

## 4. Discussion

Ensuring high array reflectivity (large |*S*_11_|) requires adequate conductivity of the liquid–metal patches, which in turn requires minimizing liquid–metal oxidation during the spray-coating process. This section discusses the effect of process variations introduced during the fabrication process—specifically, the effect of liquid–metal patch length variations due to spray-time durations and liquid–metal bulk conductivity variations due to oxidation.

### 4.1. Effect of Variations in Patch Length

A short spraying duration is desired to reduce liquid–metal oxide formation, which reduces electrical conductivity [39]. However, a shorter spraying duration also increases the chance of under-spraying. Liquid metal may not coat the target surface evenly, resulting in incomplete coverage of some areas and, therefore, uneven patch lengths *l_p_*, as previously shown in Figure 7 and Figure 8.

**Figure 14:**
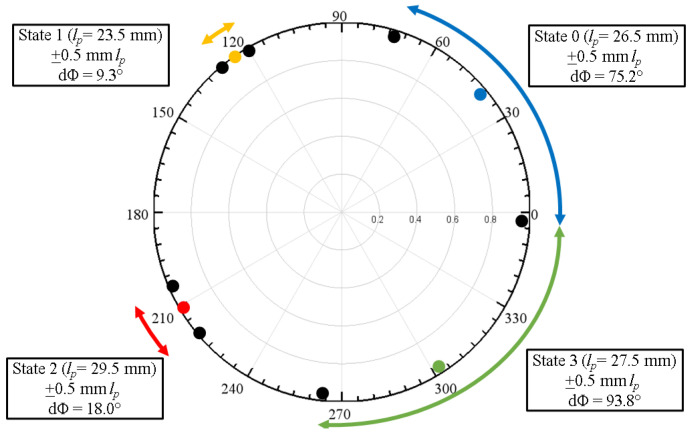
*S*_11_ of four unit-cell states at 3.46 × 10^6^ S/m as *l_p_* is varied ±0.5 mm.

**Figure 15:**
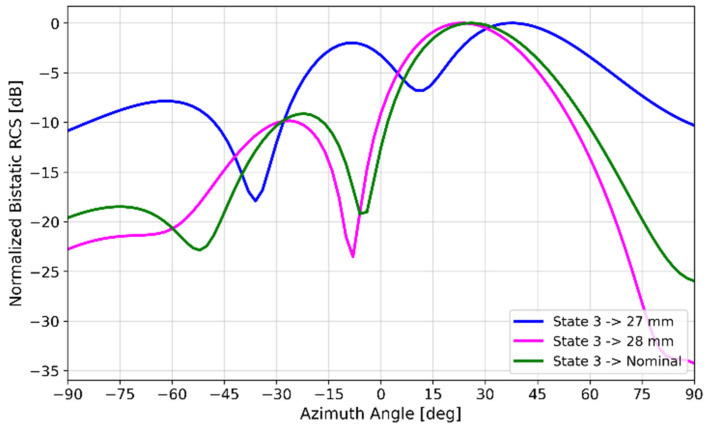
Bistatic RCS simulations of a 6 × 4 spray-coated liquid–metal metasurface varying State 3 patch length by ±0.5 mm.

Floquet-port and finite-array simulations were performed in HFSS to study the effect of variations in patch length *l_p_*, which affect unit-cell resonances due to inductance and capacitance effects [42], thereby affecting array performance. Figure 14 illustrates the change in *S_11_* if the patch lengths varied ±0.5 mm from their nominal values in Figure 3. It was observed that the effects of *l_p_* variations in all four states were not uniform, with State 0 (*l_p_* = 26.5 mm) and State 3 (*l_p_* = 27.5 mm) having the most potential to introduce error because of the wide phase variability. Figure 15 illustrates the effect of varying the lengths of all State 3 unit cells in a 6 × 4 array by ±0.5 mm from its nominal value of 27.5 mm. It is interesting to note the similarity of Figure 15 to Figure 9, where two lobes appeared instead of the well-defined lobe at 28°. While Figure 9 is a simulation of the array incorporating the as-fabricated shortened patch lengths, which predicts both the anomalous reflecting lobe at 28° as well as an emerging broadside lobe at 0°, Figure 14 provides the insight leading to this behavior: that the shortening of certain states in particular lead to a phase-gradient imbalance.

### 4.2. Effect of Variations in Liquid–Metal Bulk Conductivity

Figure 5 shows that there was a negligible difference in the bistatic RCS simulation of a Galinstan versus copper 6 × 4 array, despite the order-of-magnitude difference in conductivity (3.46 × 10^6^ S/m vs. 5.8 × 10^7^ S/m).

**Figure 16:**
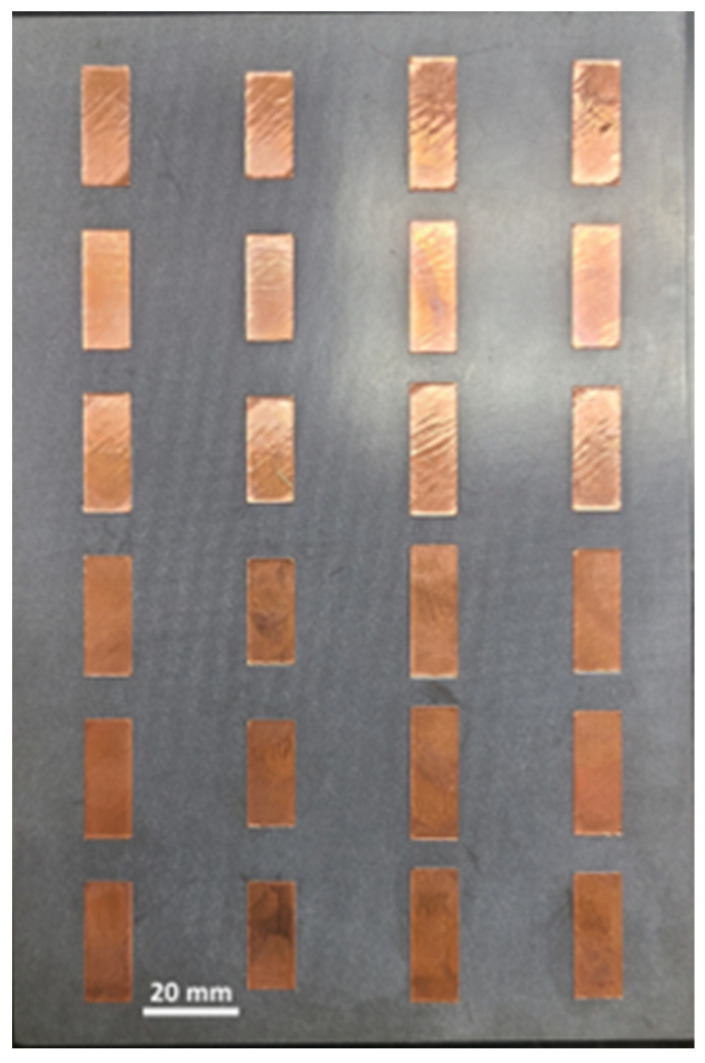
Fabricated 6 × 4 element copper metasurface to compare to the spray-coated liquid–metal metasurface in Figure 6.

Experimentally, however, we did observe a difference away from the anomalous reflection angle. For comparison purposes, a 6 × 4 copper array (Figure 16) was fabricated with a Silhouette Portrait 3 craft cutter using 0.15-mm-thick copper tape cut to the dimensions indicated in the caption of Figure 2 for the State 0-1-2-3 pattern. The detailed fabrication process is described in [43].

**Figure 17:**
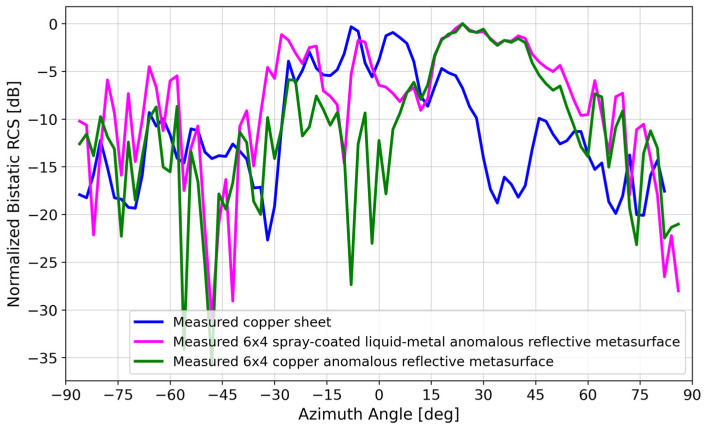
Measured bistatic RCS patterns of (i) 229 mm × 152 mm copper sheet, (ii) spray-coated liquid–metal metasurface, and (iii) static copper metasurface.

Figure 17 compares the measured bistatic RCS patterns of the copper vs. spray-coated liquid–metal metasurface. Although there was good agreement at the ~28° anomalous reflection angle, the agreement at boresight was not as good, with the spray-coated liquid–metal metasurface mimicking a copper sheet, reflecting ~10 dB more power than the copper array.

Section 4.1 discussed how patch length variations induced by pressure spraying explain some of the undesired boresight reflection effects. In addition, the radiation pattern of the metasurface can also be affected by the bulk conductivity of the reflective elements. Individual element conductivity varied depending on the amount of oxide formed due to the employed spraying parameters. In this section, we discuss liquid–metal bulk conductivity as another factor.

Liquid–metal pressure-spraying with an airbrush is characterized by three main parameters: spraying time, spraying distance, and air pump pressure [39]. In our application, air-pump pressure was constant, but due to the manual spraying procedure, spraying time and distance were variable. The liquid–metal thickness applied during the process was proportional to the spraying time and inversely proportional to the spraying distance. In addition, as the patch thickness increased, its bulk conductivity decreased due to an increased presence of oxide [39]. During longer spraying periods, the oxide layer on the liquid–metal surface was constantly broken, allowing more metal to become exposed to air. Thus, precautions must be taken to reduce the amount of air exposure to the liquid–metal patches, mainly in the form of low pressure and decreased spraying times.

**Figure 18:**
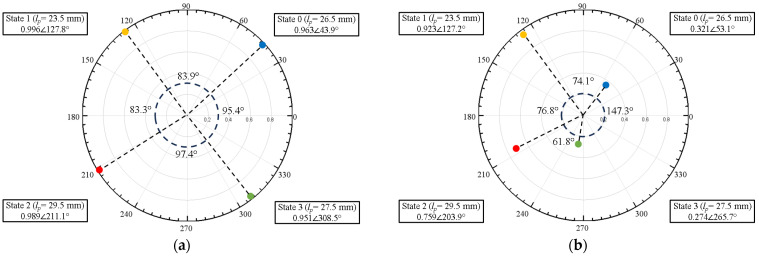
*S*_11_ of four unit-cell states at 3.5 GHz for liquid–metal conductivities of (**a**) 3.46 × 10^6^ S/m and (**b**) 1.4 × 10^4^ S/m. Figure 18a is identical to Figure 3b but is replicated here to facilitate comparison to Figure 18b.

Figure 18 illustrates the change in *S*_11_ at 3.5 GHz if the conductivity was reduced from 3.46 × 10^6^ S/m to 1.4 × 10^4^ S/m. The bulk conductivity upper-bound value was taken from [31], while the lower-bound value was from the work presented in [39], in which 1.4 × 10^4^ S/m was the smallest conductivity value measured using similar airbrush spraying parameters as our tests.

**Figure 19:**
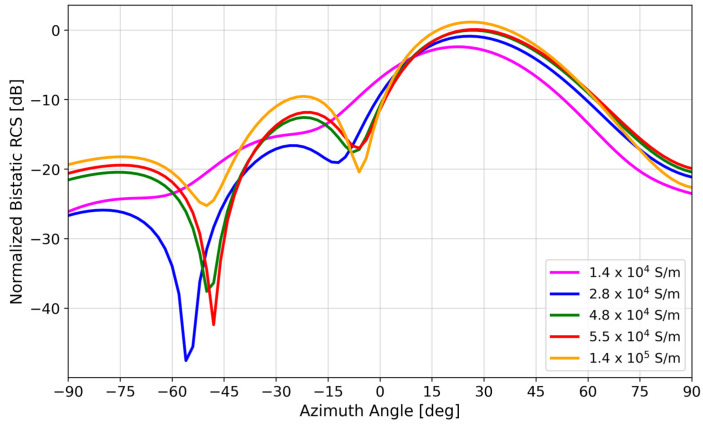
Bistatic RCS simulations of a 6 × 4 spray-coated liquid–metal metasurface using Galinstan with varying bulk conductivity. This simulation used the as-designed patch-length dimensions specified in the caption of Figure 2, so the effects of varying conductivity can be observed independent of variations in the as-fabricated patch lengths.

The most obvious effect in comparing Figure 18a,b was the |*S*_11_| reduction for States 0, 2, and 3, but interestingly, there was not too much of an effect on ∠*S*_11_, compared to the effect of patch length on ∠*S*_11_ as shown in Figure 14. This is supported by the bistatic RCS pattern of a homogeneously low-conductivity metasurface illustrated in Figure 19, in which the anomalous reflection lobe at 28° was maintained. However, due to the decreased phase gradient between adjacent unit cells, the metasurface approached that of a uniform metal sheet, with more boresight reflection and a reduced side lobe due to the reduced phase gradient along the surface.

Other factors, such as the resulting droplet size of the liquid metal due to the employed spraying parameters and the temperature of the liquid metal, could contribute to the change in bulk conductivity that results in the slight deformation of the radiation pattern in Figure 19. In combination with the effects of the variation on patch length and the amount of oxide due to the spraying parameters, the resulting mismatch between the simulated and fabricated spray-coated liquid–metal anomalous reflective metasurface seen in Figure 11 occurred.

## 5. Conclusions

This paper presented what is believed to be the first rapidly prototyped anomalous reflective metasurface based on spray-coating of Galinstan liquid metal. As a demonstration, a 6 × 4 element spray-coated liquid–metal metasurface operating at 3.5 GHz demonstrated an anomalous reflection at 28°, similar to a metasurface with copper reflective elements of the same dimensions. Differences between the measured and simulated results of the spray-coated liquid–metal metasurface were attributed to variances in the fabricated patch lengths and the bulk conductivity of the liquid–metal films. Therefore, future works could be geared toward fabrication process improvements.

Manual spray-coating was used to fabricate the liquid–metal reflective metasurface, making it susceptible to variations in patch dimensions and conductivity. Computer-controlled spray-coating would help achieve constant spraying parameters, removing unwanted variations and improving both the fabrication process and RF performance.

The liquid–metal spray-coating prototyping technique has the potential for reusability, scalability, and reconfigurability. Other prototyping methods, such as sputter deposition, inkjet printing, screen printing, and 3D printing, suffer from equipment limitations that affect the scalability and cost of the metasurface. With pre-existing masks, our proposed approach for rapid prototyping of anomalous reflective metasurfaces allows for faster fabrication with inexpensive deposition equipment. Our proposed approach also offers lower recurring costs as the masks, the substrates, and the Galinstan are potentially reusable, as Galinstan can be removed and recycled using an electrolyte solution like sodium hydroxide [44].

The performance of a metasurface is highly dependent on the scalability of the array, which is only limited by the size of the substrate and the material of the mask. To overcome the size limitation of the substrate, a tiling method could be used where multiple substrates are placed together, and the liquid metal elements are then spray-coated using a mask. The material of the mask determines its reusability, which means that a sturdier material that is compatible with liquid metal would allow a mask to be reused indefinitely, allowing for easier scalability.

With pre-existing masks and removable reflective elements, our proposed liquid–metal spray-coating technique offers potential reconfigurability of the metasurface. The reflective metasurface demonstrated in this paper was able to redirect a normal beam toward one anomalous angle. Other angles are possible by using a different template with a different coding sequence, e.g., State 0-0-2-2. Some sequences even resulted in multiple anomalous angles so that multiple response teams in Figure 1 can be linked to the mobile base station simultaneously.

## Figures and Tables

**Table 1:** Maximum directivity of liquid–metal metasurface compared to [40].

Device	Maximum Directivity [dBi]		
	Uniform Aperture [41]	Analytical Model	Measured
Pin-diode-based Copper RIS 10λ × 10λ, 27.5–29.5 GHz [40]	30.5	27.9	22.5
Passive Galinstan Metasurface 2.67λ × 1.77λ, 3.5 GHz [This paper]	17.7	16.4 (As-designed) 13.9 (As-fabricated)	13.2

## Data Availability

The data presented in this study are available upon request.

## Supplementary Materials

The following supporting information can be downloaded at https://www.mdpi.com/article/10.3390/ma17092003/s1, Table S1. As-fabricated measurements of 6 × 4 spray-coated liquid–metal anomalous reflective metasurface patch dimensions.
